# Genetic Evidence for Erythrocyte Receptor Glycophorin B Expression Levels Defining a Dominant Plasmodium falciparum Invasion Pathway into Human Erythrocytes

**DOI:** 10.1128/IAI.00074-17

**Published:** 2017-09-20

**Authors:** Selasi Dankwa, Mudit Chaand, Usheer Kanjee, Rays H. Y. Jiang, Luis V. Nobre, Jonathan M. Goldberg, Amy K. Bei, Mischka A. Moechtar, Christof Grüring, Ambroise D. Ahouidi, Daouda Ndiaye, Tandakha N. Dieye, Souleymane Mboup, Michael P. Weekes, Manoj T. Duraisingh

**Affiliations:** a Department of Immunology and Infectious Diseases, Harvard T.H. Chan School of Public Health, Boston, Massachusetts, USA; b Cambridge Institute for Medical Research, University of Cambridge, Cambridge, United Kingdom; c Laboratory of Bacteriology and Virology, Le Dantec Hospital, Cheikh Anta Diop University, Dakar, Senegal; University of South Florida

**Keywords:** Plasmodium falciparum, cultured erythrocyte, glycophorin, host cell invasion, malaria, red blood cells, transcriptional variation

## Abstract

Plasmodium falciparum, the parasite that causes the deadliest form of malaria, has evolved multiple proteins known as invasion ligands that bind to specific erythrocyte receptors to facilitate invasion of human erythrocytes. The EBA-175/glycophorin A (GPA) and Rh5/basigin ligand-receptor interactions, referred to as invasion pathways, have been the subject of intense study. In this study, we focused on the less-characterized sialic acid-containing receptors glycophorin B (GPB) and glycophorin C (GPC). Through bioinformatic analysis, we identified extensive variation in glycophorin B (GYPB) transcript levels in individuals from Benin, suggesting selection from malaria pressure. To elucidate the importance of the GPB and GPC receptors relative to the well-described EBA-175/GPA invasion pathway, we used an *ex vivo* erythrocyte culture system to decrease expression of GPA, GPB, or GPC via lentiviral short hairpin RNA transduction of erythroid progenitor cells, with global surface proteomic profiling. We assessed the efficiency of parasite invasion into knockdown cells using a panel of wild-type P. falciparum laboratory strains and invasion ligand knockout lines, as well as P. falciparum Senegalese clinical isolates and a short-term-culture-adapted strain. For this, we optimized an invasion assay suitable for use with small numbers of erythrocytes. We found that all laboratory strains and the majority of field strains tested were dependent on GPB expression level for invasion. The collective data suggest that the GPA and GPB receptors are of greater importance than the GPC receptor, supporting a hierarchy of erythrocyte receptor usage in P. falciparum.

## INTRODUCTION

Malaria is a disease of major global health importance that is caused by parasites of the genus Plasmodium, of which Plasmodium falciparum is the most virulent (1). The asexual erythrocytic stage of the parasite life cycle is responsible for the symptoms associated with malaria (1). A key step during this stage is parasite invasion of erythrocytes mediated by interactions between parasite invasion ligands and their cognate erythrocyte receptors, which define invasion pathways. Across Plasmodium species, invasion ligands are grouped into two families: the Duffy binding-like or erythrocyte binding-like (DBL/EBL) family and the reticulocyte binding-like (RBL) family (2–4). P. falciparum has four EBL ligands, i.e., EBA-175, EBL-1, EBA-140 (BAEBL), and EBA-181 (JESEBL), and five RBL ligands, i.e., Rh1, Rh2a, Rh2b, Rh4, and Rh5. Of these, the cognate receptors of five invasion ligands are known: glycophorin A (GPA), GPB, and GPC, which bind EBA-175 (5), EBL-1 (6, 7), and EBA-140 (8, 9), respectively, and complement receptor 1 (CR1) and basigin (BSG), which are receptors for Rh4 (10, 11) and Rh5 (12), respectively.

Invasion pathways can be classified according to their dependence on the presence of sialic acid on receptors; pathways involving the EBL invasion ligands and Rh1 are reliant on sialic acid (sialic acid-dependent invasion pathways), while those involving the remaining RBL ligands are not (sialic acid-independent invasion pathways). Although P. falciparum has an extensive array of invasion pathways, not all are utilized at the same time. The set of dominant invasion pathways used during invasion is strain dependent and has led to a broad classification of P. falciparum strains as sialic acid dependent or independent. However, laboratory-adapted strains have the ability to switch invasion pathway usage when specific receptors or determinants of interaction are absent from the erythrocyte surface (13, 14). Furthermore, field isolates commonly utilize different sets of invasion pathways (15–19). The virulence of P. falciparum has been partly attributed to its extensive set of invasion pathways, which enable it to efficiently invade diverse host erythrocytes harboring different receptor polymorphisms.

Most recently, the Rh5/BSG sialic acid-independent invasion pathway has received the greatest attention owing to the essentiality of the Rh5/BSG invasion pathway (12, 20). Other studies have also shown that the EBA-175/GPA sialic acid-dependent invasion pathway plays a significant role in both sialic acid-dependent and sialic acid-independent strains (21–23). Importantly, naturally occurring anti-EBA-175 and anti-Rh5 invasion-inhibitory antibodies have been identified in individuals exposed to malaria (24–27).

Less characterized are the EBA-140/GPC sialic acid-dependent invasion pathway (8, 9, 28, 29) and the sialic acid-dependent parasite invasion ligand EBA-181, for which no receptor has been identified (30–32). The EBL-1/GPB invasion pathway has also been poorly characterized, and there are contradictory reports regarding the importance of GPB. One study reported a complete block in invasion of the sialic acid-independent strain 7G8 into GPB-null (S-s-U-) erythrocytes (33), although a prior study showed little inhibition of this strain (34). A subsequent study showed a 40 to 87% range in efficiency of invasion into S-s-U- erythrocytes from five donors (35). Donor-to-donor blood group differences and differences in receptors may contribute to the variable invasion phenotypes of GPB-null cells, which underscores a potential weakness of comparing nonisogenic mutant and wild-type erythrocytes.

In a search for novel signatures of P. falciparum infection, we performed computational analysis of a published data set of transcriptomic profiles from malaria-infected and healthy Beninese children (36), which led to the discovery that there is wide variation in glycophorin B transcript (GYPB) transcript levels in healthy individuals. This finding provided the impetus for a detailed study of the use of the GPB receptor in P. falciparum invasion. In this study, we used an erythrocyte reverse genetics system (37) to specifically knock down levels of expression of the sialic acid-dependent receptors GPA, GPB, and GPC. We report that GPB is a key determinant of P. falciparum invasion.

## RESULTS

### GYPB transcript levels vary widely among healthy individuals in a region where malaria is endemic.

**FIG 1:**
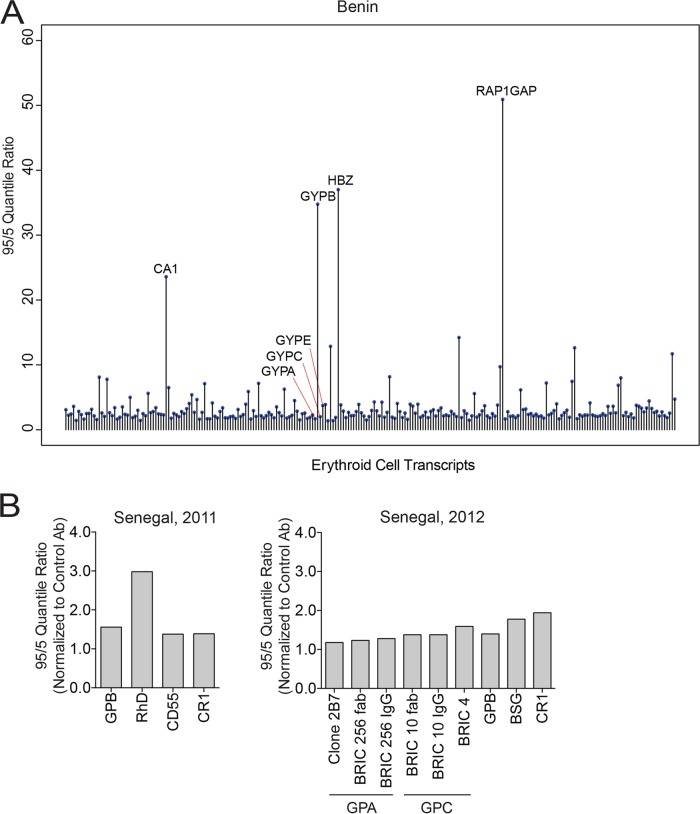
Glycophorin B transcript level variation and erythrocyte receptor expression among individuals in regions where malaria is endemic. (A) Graph highlighting transcripts, including GYPB, whose abundance varied widely among erythroid cell-specific transcripts from 61 healthy individuals in Benin. Bioinformatic analysis was based on the whole blood transcriptomics study of malaria-infected and healthy children in Benin (36). (B) Expression of GPB and other erythrocyte receptors from blood of healthy Senegalese donors in 2011 (left graph) and 2012 (right graph). The graphs show the 95/5 quantile ratio normalized to the quantile ratio for a control secondary antibody (Ab). The quantile ratio was based on mean fluorescence intensity values determined by flow cytometry. In 2011, measurements were made on RBCs from 29 donors (GPB), 32 donors (CD55 and CR1), or 41 donors (RhD), and in 2012, they were made on RBCs from 11 donors (GPB) or 29 donors (all other receptors). In 2011, one GPB-null individual and three RhD-null individuals were observed but were excluded from measurement of the 95/5 quantile ratio. In 2012, three different probes were used to measure expression of GPA and GPC (see Materials and Methods).

Erythrocyte receptors involved in P. falciparum invasion and their regulatory regions harbor polymorphisms, some of which are overrepresented in regions where malaria is endemic and are suggested to have arisen as a consequence of the selective force of malaria on the human genome. To determine if we could identify additional polymorphisms that might affect P. falciparum infection, we performed computational analysis of transcriptional profiles generated from whole blood of children in Benin, published by Idaghdour et al. (36). We first defined a subset of erythroid cell-specific genes from the transcriptome based on the HaemAtlas published by the Bloodomics Consortium (38) and from known blood groups. Of 238 erythroid cell-specific/blood group transcripts from 61 healthy children included in the analysis, we identified four genes with wide expression ranges (95 quantile to 5 quantile ratio larger than 10 and z score greater than 3): those for carbonic anhydrase 1 (CA1), hemoglobin zeta chain (HBZ), RAP1 GTPase-activating protein 1 (RAP1GAP), and, unexpectedly, GYPB (Fig. 1A). Importantly, many transcripts with little variation served as internal controls (Fig. 1A).

To determine if this GYPB transcript level variation translated to receptor expression variation, we measured the levels of GPB, as well as GPA, GPC, and other blood group receptors, in healthy donors in Senegal, where malaria is endemic, over two consecutive years (Fig. 1B). The variation in surface expression of GPB in GPB-positive individuals, estimated by the 95/5 quantile ratio, was ∼1.5-fold above background, much smaller than the transcript level variation found in the Beninese children. We also observed modest variation in surface expression of other receptors we measured, with a 95/5 quantile ratio ranging from ∼1.2 to 3 (Fig. 1B).

### Knockdown of GPA, GPB, or GPC in *ex vivo* cRBCs.

**FIG 2:**
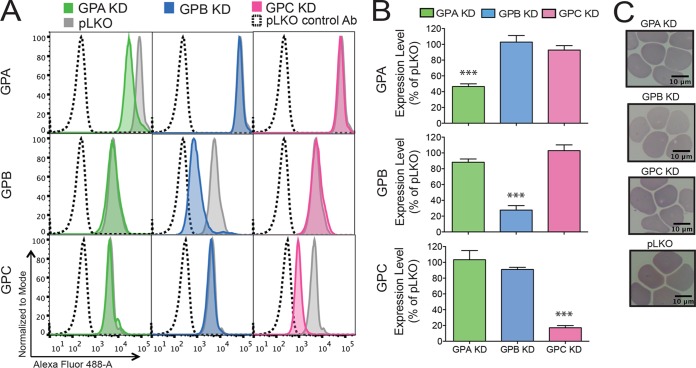
Knockdown of glycophorins A, B, and C in cultured erythrocytes. (A) Expression of glycophorin A (GPA), glycophorin B (GPB), and glycophorin C (GPC) on the cell surface of GPA knockdown (KD) (green), GPB KD (blue), and GPC KD (pink) cultured erythrocytes (cRBCs) as determined by flow cytometry. Representative flow cytometry plots are shown. Gray traces, receptor expression on pLKO cRBCs; dashed traces, pLKO cells stained with a control antibody. (B) Mean expression ± standard error from three to seven independent experiments, normalized to expression on pLKO cells. For measurement of GPB expression, cRBCs were treated with trypsin to remove GPA and cells stained with a GPA/B antibody. Statistical significance was determined using a one-way analysis of variance (ANOVA) with a Dunnett multiple-comparison test. ***, *P* ≤ 0.001. (C) May-Grünwald- and Giemsa-stained cytospins showing normal morphology of KD and pLKO control cRBCs. cRBCs were passed through a 5-μm filter to remove nucleated cells prior to flow cytometry and cytospin preparation.

To understand expression level modulation of GPB in P. falciparum invasion and to determine the importance of this receptor relative to the other major sialic acid-dependent receptors, GPA and GPC, we knocked down expression of GPB in *ex vivo* cultured erythrocytes (cRBCs) via short hairpin RNA (shRNA)-mediated gene silencing (37). In 6 or 7 independent experiments, we achieved approximately 53.5% ± 8.9% (mean ± standard deviation [SD]) knockdown (KD) of GPA (*P* ≤ 0.001), 72.3% ± 14.2% KD of GPB (*P* ≤ 0.001), and 82.9% ± 6.5% KD of GPC (*P* ≤ 0.001) (Fig. 2A and B), as determined by flow cytometry. We also measured the expression levels of all of the glycophorins (between three and seven times) following KD of each receptor and found little change in the expression levels of the others by flow cytometry (Fig. 2B). We further measured surface expression by flow cytometry of other known Plasmodium receptors (BSG, band 3, CR1, and DARC) (see Fig. S1 in the supplemental material). We observed a 4-fold increase in band 3 levels following GPA KD, as previously reported (39), a 2-fold increase in BSG levels on GPA KD cells, and an ∼25% decrease in BSG levels on GPB KD cells. Further, we found no association between GPB and BSG levels by flow cytometry in Senegalese individuals (Fig. 1B). In addition, KD and pLKO control cRBCs had normal erythrocyte morphology (Fig. 2C).

### Global proteomic profiling to determine the level and specificity of GPA, GPB, and GPC receptor knockdowns.

**FIG 3:**
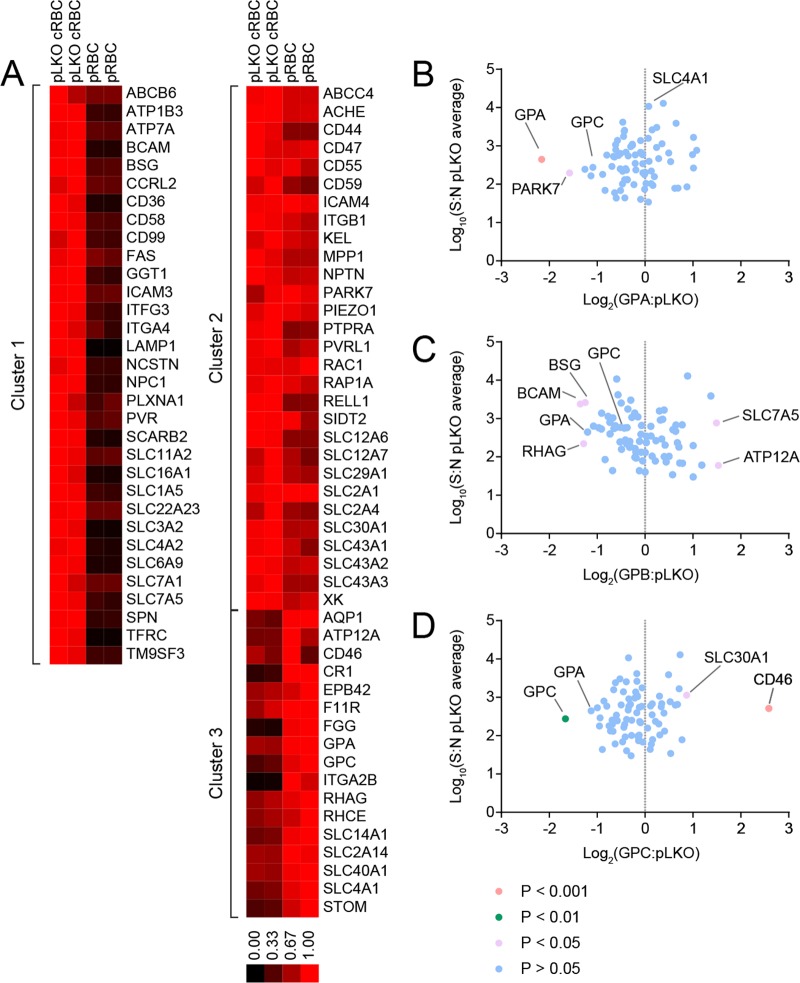
Quantitative cell surface proteomics of peripheral erythrocytes and cultured erythrocytes. (A) Quantitative cell surface proteomic comparison between pLKO cRBCs and peripheral RBCs (pRBCs). k-means clustering analysis of the 78 surface membrane proteins identified three clusters: cluster 1, proteins that decrease between pLKO cRBCs and pRBCs; cluster 2, proteins that remain at the same level between pLKO cRBCs and pRBCs; and cluster 3, proteins that increase between pLKO cRBCs and pRBCs. The scale bar represents the normalized relative abundance of each protein. Note that the peptides detected by this technique did not allow a distinction to be made between GPA and GPB. (B to D) Comparison of the relative abundances of membrane proteins between the pLKO control cRBCs and either the GPA KD cRBCs (B), GPB KD cRBCs (C), or GPC KD cRBCs (D). Fold change was calculated as the ratio of the signal/noise ratio for GPA/B/C KD) to the average signal/noise for the pLKO control. *y* axes show the average signal/noise (S:N) ratio across all samples. *P* values were estimated using Benjamini-Hochberg-corrected significance A, calculated in Perseus v 1.5.1.6.

To establish the similarity between cRBCs and physiologically derived red blood cells (pRBCs), we measured expression of 78 cell surface receptors by plasma membrane profiling (PMP) on cRBCs and pRBCs (see Table S1 in the supplemental material). First, this analysis demonstrated the reticulocyte-like nature of the cRBCs by showing that several proteins that decrease in abundance during reticulocyte maturation, including CD71 (TFRC) (40, 41), CD36 (42, 43), ITGA4 (43), and SLC3A2 (44), were decreased in pRBCs relative to pLKO cRBCs (cluster 1) (Fig. 3A). The level of expression of proteins in cluster 2 remained unchanged between pRBCs and pLKO cRBCs, while cluster 3 proteins were enriched on pRBCs and included GPA and GPC. (By this method, we are unable to distinguish between GPA and GPB peptides; thus, due to the ∼4-fold-greater abundance of GPA receptors, we assume that the signal emanates from a GPA/GPB ratio of 4:1.)

We have calculated the numbers of molecules of known P. falciparum host receptors per unit surface area (45–48, 50–52) (Table 1), using published values of the surface areas of reticulocytes (142.4 ± 2.0 μm^2^) and pRBCs (133.6 ± 3.0 μm^2^) (45). We find that the surface densities of receptors on pLKO cells and pRBCs for all known receptors are within 3-fold of each other, except for CR1, whose density is ∼5-fold lower on pLKO cRBCs. GPA/GPB and GPC are found at lower levels on pLKO cells, while BSG is the only major receptor that is found at a higher level. The lower density of GPA/GPB and GPC on pLKO cells suggests that further limitation of receptor densities by knockdown will elicit receptor densities significantly lower than those observed on pRBCs (Table 1).

**TABLE 1:** Comparison of copies of known P. falciparum host receptors per unit surface area on peripheral erythrocytes and cultured erythrocytes^*a*^

Protein (reference[s])	No. of copies/μm on erythrocyte type:				
	pRBC	pLKO	GPA KD	GPB KD	GPC KD
GPA (48, 50, 52)	7.49 × 10^3^	4.42 × 10^3^	1.25 × 10^3^	1.62 × 10^3^	2.38 × 10^3^
GPB (48, 50, 52)	1.87 × 10^3^	1.11 × 10^3^	3.11 × 10^2^	4.05 × 10^2^	5.95 × 10^2^
GPC (48, 50, 51)	7.49 × 10^2^	2.57 × 10^2^	1.52 × 10^2^	1.62 × 10^2^	9.51 × 10^1^
BSG (47, 50)	2.25 × 10^1^	6.26 × 10^1^	8.72 × 10^1^	2.21 × 10^1^	6.52 × 10^1^
CR1 (50)	(1.50–8.98) × 10^0^	(0.31–1.84) × 10^0^	(0.41–2.43) × 10^0^	(0.38–2.31) × 10^0^	(0.52–3.13) × 10^0^
Band 3 (47)	7.49 × 10^3^	3.53 × 10^3^	4.68 × 10^3^	1.97 × 10^3^	3.26 × 10^3^
Kx (47)	7.49 × 10^0^	8.81 × 10^0^	1.05 × 10^1^	7.04 × 10^0^	8.36 × 10^0^
CD55 (47)	1.50 × 10^2^	1.59 × 10^2^	1.49 × 10^2^	9.47 × 10^1^	1.42 × 10^2^
CD44 (46, 47)	2.62 × 10^1^	4.47 × 10^1^	6.66 × 10^1^	3.05 × 10^1^	3.64 × 10^1^

a The reported numbers of copies of known P. falciparum host receptors on peripheral erythrocytes (pRBCs) were used to estimate the numbers of copies of host receptors on pLKO cRBCs based on the normalized signal/noise ratios from quantitative surface proteomics and the following reported surface areas for mature RBCs of 133.6 ± 3.0 μm^2^ and for reticulocytes of 142.4 ± 2.0 μm^2^ (45), which we assume to be representative of pLKO cRBCs. Since GPA and GPB peptides are indistinguishable by surface proteomics, estimation was based on the reported relative abundance of GPA and GPB on pRBCs. The densities of host receptors per unit surface area for GPA, GPB, and GPC are within 3-fold lower on pLKO cRBCs than on pRBCs.

To ensure that the specificity of invasion phenotypes would result specifically from the receptors that were depleted, we made a comparison of pLKO and KD cRBCs. As expected, GPA and GPC were reduced in the GPA and GPC KD cRBCs, respectively (Fig. 3B and D). As mentioned above, GPA and GPB peptides are indistinguishable; therefore, GPB depletion in the GPB KD cells was not observable by this analysis. Of all the known receptors considered, only BSG levels were reduced (albeit at borderline significance [*P* = 0.047]) (Table S1 and Fig. 3C) in GPB KD cells. Calculated surface densities of BSG on GPB KD cells are comparable to those found on pRBCs (Table 1), suggesting that BSG is not limited to levels that would contribute to an invasion phenotype. This analysis also revealed other proteins that have significantly altered levels in pLKO cRBCs relative to KD cRBCs (Fig. 3B to D; see Table S2 in the supplemental material). To date, no role in invasion has been described for these proteins.

Interestingly, band 3 (SLC4A1) levels were not significantly elevated by proteomic analysis on GPA KD cells (Fig. 3B), in contrast to the significant increase in band 3 levels determined by flow cytometry (Fig. S1) (37). One potential explanation for the discrepancy is that knockdown of GPA (known to be in a complex with band 3 [53, 54]) improves accessibility of the band 3 antibody to its epitope during flow cytometry measurements.

### A scaled-down assay for measuring erythrocyte invasion efficiency.

**FIG 4:**
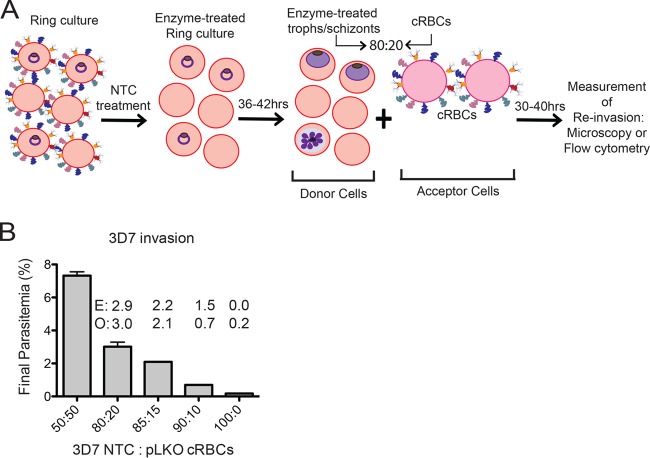
Schematic of invasion assay design. (A) A ring-stage parasite culture is treated with neuraminidase, trypsin, and chymotrypsin (NTC), returned to culture conditions, and allowed to mature to the late trophozoite (troph) or schizont stage. The treated culture (donor cells) is mixed in an 80:20 ratio with either knockdown (KD) or control pLKO cRBCs (acceptor cells). Initial parasitemia and/or final parasitemia after one round of invasion are determined either by microscopy from counts of 500 to 2,000 erythrocytes or by flow cytometry of SYBR green I-stained cells. Invasion assays are set up at 0.5% hematocrit. All acceptor cells are counted by hemocytometer prior to assay setup. (B) Invasion of P. falciparum 3D7 treated with neuraminidase, trypsin, and chymotrypsin into pLKO control cRBCs with various ratio of enzyme-treated donor cells to pLKO acceptor cells. A 100:0 ratio indicates treated 3D7 donor cells and no pLKO cRBCs. Initial parasitemia was ∼2%. Final parasitemia was determined by flow cytometry of SYBR green I-stained cells. The assay was performed once in duplicate. Bars represent the mean ± the range. The 80:20 ratio was selected for subsequent invasion assays. E, expected parasitemia based on invasion into a 50:50 mixture. O, observed parasitemia.

To enable us to assess the invasion profiles of as many P. falciparum strains as possible, we developed a scaled-down invasion assay that makes use of small numbers of cells of interest (Fig. 4A). In our assay design, a P. falciparum culture treated with neuraminidase (Nm), trypsin, and chymotrypsin, constituting donor cells, is added to cells of interest (acceptor cells) in an 80:20 ratio, and parasitemia is determined after a single round of invasion. We used the 80:20 ratio of donor to acceptor cells over other ratios because the 80:20 ratio allowed us to use a limited number of cultured erythrocytes yet gave us a reasonable, measurable final parasitemia of 3% when assessing invasion of 3D7 into pLKO control cells with a starting parasitemia of ∼2% (Fig. 4B). The success of this scaled-down assay depends on effective treatment with neuraminidase, trypsin, and chymotrypsin to prevent reinvasion into donor cells, careful counting of acceptor cells (achieved with a hemocytometer), and a reasonable starting parasitemia in the donor culture (ideally 2.5%), since only 20% of the cells in each sample have intact receptors for invasion.

### GPB is a major receptor used by P. falciparum laboratory strains.

To assess the relative importance of GPB in invasion, we tested invasion of KD cRBCs by a panel of wild-type P. falciparum laboratory-adapted strains and invasion ligand deletion strains. Using our scaled-down invasion assay, we observed decreased invasion of the sialic acid-dependent strain Dd2 into GPB KD cRBCs (56.4% ± 24.2% [mean ± SD]) as well as GPA KD cRBCs (72.9% ± 18.3%), as previously reported (37), but no change in invasion of GPC KD cells (91.8% ± 8.2%) relative to pLKO control cells (Table 2).

**TABLE 2:** Efficiencies of invasion of P. falciparum laboratory-adapted strains and a Senegalese culture-adapted isolate into knockdown cultured erythrocytes

Strain	% efficiency of invasion^*a*^ into erythrocyte type:		
	GPA KD	GPB KD	GPC KD
Dd2	72.9 ± 18.3*	56.4 ± 24.2**	91.8 ± 8.2
3D7	90.2 ± 11.4	66.6 ± 23.3***	89.6 ± 9.8
3D7ΔEBA175	100.4 ± 31.8	53.4 ± 30.0	94.1 ± 16.5
3D7ΔRh2b	101.2 ± 14.8	60.5 ± 26.6*	100.2 ± 4.8
7G8	85.0 ± 31.5	56.1 ± 26.8	98.3 ± 21.9
HB3	101.3 ± 43.7	53.6 ± 18.1	93.0 ± 15.5
Sen51	109.7 ± 28.3	58.8 ± 16.8*	113.0 ± 3.6

a Efficiency of invasion into KD cells relative to pLKO control cells, based on final parasitemia. Parasitemia was determined by microscopy from counts of 500 to 2,000 erythrocytes, depending on the experiment. Invasion assays were performed in triplicate, four to six times for 3D7, Dd2, 7G8, and HB3 and three times for 3D7ΔEBA-175, 3D7ΔRh2b, and Sen51. Shown are the means and standard deviations. Statistical significance was determined using a one-way ANOVA with a Dunnett multiple-comparison test (*, *P* < 0.05; **, *P* ≤ 0.01; and ***, *P* ≤ 0.001).

It has been shown that strain 3D7 uses the sialic acid-dependent EBA-175/GPA invasion pathway to some extent (23). We did not observe a decrease in invasion of 3D7 into GPA KD cRBCs, consistent with a previous report (37); however, the parent 3D7 strain and the derived invasion ligand deletion lines, 3D7ΔEBA-175 and 3D7ΔRh2b, had decreased invasion into GPB KD cRBCs (Table 2). This decrease reached statistical significance for 3D7 (66.6% ± 23.3%) and 3D7ΔRh2b (60.5% ± 26.6%), indicating that in 3D7, the GPB receptor is more important than the GPA receptor. None of the 3D7 strains showed decreased invasion into GPC KD cRBCs (Table 2), suggesting a lesser role in invasion for this receptor.

Like 3D7, the sialic acid-independent strains 7G8 and HB3 showed decreased invasion of GPB KD cells, though this decrease was not statistically significant (Table 2). We did not observe decreased invasion of 7G8 or HB3 into GPA or GPC KD cRBCs relative to pLKO cRBCs. Altogether, our data suggest that GPB is important for invasion by the tested sialic acid-dependent and sialic acid-independent laboratory strains of P. falciparum.

### Many P. falciparum field isolates use GPB for invasion.

To determine whether GPB is important in invasion by field isolates, we assessed invasion of a culture-adapted Senegalese isolate and fresh clinical isolates from Senegal into KD cRBCs (Tables 2 and 3). Sen51, a short-term culture-adapted strain which was sensitive to Nm treatment (defined as efficiency of invasion of less than 60% into Nm-treated pRBCs that are devoid of sialic acid [see Table S3 in the supplemental material]), showed decreased invasion into GPB KD cRBCs (58.8% ± 16.8% [mean ± SD]) but not into GPA KD cRBCs (109.7% ± 28.3%) or GPC KD cRBCs (113.0% ± 3.6%) (Table 2).

**TABLE 3:** Efficiency of invasion of P. falciparum ex vivo field isolates into knockdown cultured erythrocytes

Strain	% efficiency of invasion^*a*^ into erythrocyte type:		
	GPA KD	GPB KD	GPC KD
Th266	88.7 ± 12.1	66.2 ± 14.2	52.5 ± 16.4
Th268	100.9 ± 28.1	79.1 ± 17.8	54.8 ± 9.4
Th275	110.4 ± 60.7	118.0 ± 66.4	70.9 ± 10.0
Th303	39.0 ± 4.1	55.6 ± 4.6	69.1 ± 2.5
Th304	40.7 ± 1.6	56.0 ± 4.7	92.3 ± 24.9
Th305	30.6 ± 0	36.7 ± 5.8	86.7 ± 10.1
Th306	44.9 ± 0.1	47.6 ± 16.5	93.5 ± 42.2
Th312	28.5 ± 3.5	51.7 ± 10.1	91.8 ± 2.0

a Efficiency of invasion into KD cells relative to pLKO control cells, based on final parasitemia. Parasitemia was determined by microscopy from counts of 800 to 1,000 erythrocytes. Invasion assays were performed once, in triplicate (Th266, Th268, and Th275) or duplicate (Th303, Th304, Th305, Th306, and Th312). Errors indicate the standard deviation (Th266, Th268, and Th275) or the range (Th303, Th304, Th305, Th306, and Th312).

For invasion by fresh clinical isolates, five out of eight strains had less than 60% invasion into GPB KD cRBCs as well as GPA KD cRBCs, while none of the five had less than 60% invasion into GPC KD cRBCs relative to pLKO cRBCs (Table 3). Of these five isolates, three were sensitive to Nm-treated pRBCs (Th303, Th305, and Th306), while two were resistant (Th304 and Th312). Two other isolates which had decreased invasion only into GPC KD cRBCs were sensitive to Nm-treated pRBCs (Th266 and Th268), bringing the proportion of Nm-sensitive strains to 75% (six out of eight strains). Invasion into GPB KD cells ranged from 36.7% ± 5.8% to 118.0% ± 66.4%, while the range for GPA KD cells was 28.5% ± 3.5% to 110.4% ± 60.7% and that for GPC KD cells was 52.5% ± 16.4% to 93.5% ± 42.2% (Table 3). Overall, our results indicate that for field isolates, GPB is of comparable importance in invasion as GPA and is more utilized than GPC for most strains tested, though there is more heterogeneity in receptor usage for field isolates than for lab lines. Furthermore, there is not a simple relationship between sialic acid dependence and the phenotype of invasion into KD cRBCs.

## DISCUSSION

In this study, we have used erythrocyte reverse genetics in an isogenic background to specifically and comparatively assess the use of three major sialylated erythrocyte receptors, GPA, GPB, and GPC, in invasion of erythrocytes by P. falciparum laboratory-adapted and field strains. We used genetically altered cRBCs to evaluate the contribution of specific invasion ligands in isogenic cells in *ex vivo* parasite invasion assays. This contrasts with previous studies, which depended on enzyme-treated cells and nonisogenic naturally occurring mutant erythrocytes to reveal invasion pathways (39). The cRBCs used in this study express the full complement of P. falciparum receptors and are of a relatively homogeneous age, though their reticulocyte-like nature results in some differences in surface receptor abundance relative to that of pRBCs. Nevertheless, we achieved limiting numbers of receptors on KD cells, enabling us to determine the relative importance of specific receptors. In comparing invasion into GPA, GPB, and GPC KD cells, we observed a dependence on GPB by both sialic acid-dependent and sialic acid-independent laboratory strains; this reliance on GPB was greater than that on GPA, which is used by many laboratory strains regardless of sialic acid dependence (21, 23). Our data also suggest that the GPC receptor is less important for invasion in most strains tested.

Bioinformatic analysis of transcriptional profiles of Beninese children revealed a wide variation in GYPB transcript levels, and one factor that might contribute to this is *GYPB* genetic polymorphisms in Africans. A high prevalence of the GPB-null genotype exists in regions where malaria is endemic and among individuals of African descent (ranging from 2 to 8% in West Africa to as high as 59% among the Efe pygmies in the Democratic Republic of Congo [55, 56]), suggesting that malaria pressure selected for this polymorphism. The Dantu variant of GPB, representing a hybrid GPA-GPB molecule with a GPB N-terminal region and a GPA C-terminal region, has been shown to confer protection against invasion and growth of P. falciparum *in vitro* (57). A recent study has found evidence for a strong protective effect of the Dantu NE genotype against cerebral malaria and severe malaria anemia in East Africa (56). The Dantu NE genotype, which results from an intricate structural modification of the *GYPE-GYPB-GYPA* locus, including deletion of the 3′ end of *GYPB*, was found to have arisen recently in Kenya. In addition to the Dantu variant, those authors identified multiple examples of deletions and duplications at the *GYPE-GYPB-GYPA* locus. In our study, we did not observe variation in GPB surface expression in healthy Senegalese to the same extent as that seen for transcript levels in Beninese individuals, suggesting that GYPB transcript level differences do not directly reflect surface receptor expression. Alternatively, it is possible that there are country-specific differences in GYPB polymorphisms, such that Beninese but not Senegalese individuals exhibit GYPB transcript level variation with concomitant variation in surface expression levels. Further investigation is required to understand the origin and significance of GYPB variation.

The observation from this work that GPB is an important receptor for P. falciparum invasion is at odds with work showing that some laboratory-adapted strains (58, 59) and field isolates from Kenya (60) and Columbia and Peru (61) have either an *ebl-1* gene deletion, a thymidine insertion, or a premature stop codon that results in a truncated EBL-1 product. Prior to these findings, some of the studies that had reported use of the EBL-1/GPB invasion pathway had used P. falciparum strains that have a mutated or deleted *ebl-1* gene, for example, 7G8, 3D7, or HB3 (33, 35), suggesting that perhaps there is an additional parasite ligand that binds to GPB. In this study, we observed usage of GPB by laboratory strains that are reported to lack a functional EBL-1 ligand, i.e., 7G8 and 3D7 (59) and HB3 (58), further suggesting that there is an additional parasite ligand for GPB. Such a ligand may have features in common with the other P. falciparum invasion ligands that bind glycophorins (EBA-175, EBL-1, and EBA-140), such as a DBL-like domain.

Importantly, our work demonstrates that many field strains use GPB for invasion. Given this dependence on GPB in a region with GPB-null prevalence, it would be relevant to genotype field isolates to determine if there are any mutations in *ebl-1*, as has been noted in some field isolates in Kenya (60) and Columbia and Peru (61), and in the absence of inactivating mutations, to determine whether invasion-inhibitory EBL-1 antibodies exist in the Senegalese population. Identifying an alternate invasion ligand that binds to GPB would warrant assessing usage by field isolates and determining the ability of naturally acquired antibodies against this ligand to block invasion, which may lead to consideration of this ligand for inclusion in an invasion-blocking subunit vaccine.

Eight of the nine field strains in this study showed decreased invasion into GPA, GPB, and/or GPC KD cells, emphasizing the sialic acid-dependent nature of many Senegalese strains. We also found that several field isolates had decreased invasion into Nm-treated pRBCs, consistent with previous field studies reporting decreased invasion of Nm-treated pRBCs (15, 18, 26, 62). However, we did not find a simple concordance between decreased invasion into GPA, GPB, and GPC KD cRBCs and sialic acid dependence (as determined by invasion into Nm-treated pRBCs) (see Table S3 in the supplemental material), underscoring the importance of separately assessing specific receptors. In this study, we have investigated the use of three major sialylated receptors and in so doing have highlighted the lesser role of GPC compared with GPA or GPB. The minor role of the EBA-140/GPC invasion pathway, especially in laboratory strains, is in concordance with other studies (21, 23). Knockout of the EBA-140 invasion ligand is facile in all laboratory strains (9), and in contrast to knockouts of EBA-175, it does not lead to a change in the use of ligand-receptor interactions (23). Jiang et al. showed that chymotrypsin treatment of GPA-null cells [En(a−)] causes an almost complete block in invasion by 10 P. falciparum laboratory strains (21). Both the EBA-175/GPA and the EBA-140/GPC invasion pathways are chymotrypsin resistant, indicating minimal use of the EBA-140/GPC invasion pathway in the absence of GPA (21). In addition, the limited requirement of GPC for erythrocyte invasion can be viewed in light of the interaction between the subtelomeric variant open reading frame protein (STEVOR) and GPC (63) for the rosetting of parasite-infected erythrocytes, suggesting an additional role for this erythrocyte receptor.

Recent studies have provided more evidence to demonstrate the importance of deformability in the invasion process (64, 65). We did not measure deformability of KD cRBCs; however, it is unlikely that changes in deformability of cRBCs as a result of receptor KDs account for the phenotypes of invasion of P. falciparum strains into KD cRBCs. This is because GPA-null cells and GPB-null cells have deformability and membrane mechanical stability similar to those of wild-type pRBCs (66), whereas GPC-null cells reportedly have decreased membrane stability and deformability (66). However, the majority of P. falciparum strains in this study had normal invasion into GPC KD cRBCs.

Altogether, the genetic evidence presented in this study reveals that the GPB invasion pathway is important for invasion by the strains tested. Our study provides the impetus for a more detailed investigation into the use of GPB by alternative invasion ligands and the use of EBL-1 in invasion by field isolates, both of which could contribute to the dominance of GPB as a receptor in the hierarchy of the RBL and EBL invasion ligands.

## MATERIALS AND METHODS

### Bioinformatic analyses.

A set of 314 host genes with known or potential roles in parasite invasion was defined by combining erythroblast-specific genes from HaemAtlas (38) with blood group genes from the International Society of Blood Transfusion (http://www.isbtweb.org/). Whole-blood raw transcriptomic data from 61 healthy Beninese children (36) were obtained from the Gene Expression Omnibus (GSE34404). Two hundred thirty-eight genes from the erythroblast-specific/blood group set defined above are expressed in the Benin data set. This subset was background corrected using the detection *P* value and quantile normalized using limma (67). The 95th to 5th quantile ratios were calculated to identify the most variable transcripts.

### *Ex vivo* culture of erythrocytes.

A total of 3 × 10^5^ to 10 × 10^5^ bone marrow-derived CD34^+^ hematopoietic stem cells (HSCs) (Lonza) or CD34^+^ cells derived from peripheral blood of granulocyte colony-stimulating factor (G-CSF)-stimulated donors (obtained from the HSCI-Boston Children's Hospital FACS Core) were cultured in Iscove's modified Dulbecco's medium (IMDM) (Biochrom) supplemented with glutamine (Sigma-Aldrich), holo-human transferrin (Scipac), recombinant human insulin (Sigma-Aldrich), heparin Choay (USBioAnalyzed), and 5% solvent/detergent virus-inactivated plasma (Octaplas; Octapharma) as described previously (68) with the following modifications. On day 6 or 7, cells were transduced with lentivirus harboring short hairpin RNA (shRNA) against GYPA (clone TRCN0000116453), GYPB (clone TRCN0000084081), or GYPC (clone TRCN0000083398) or with the empty vector (pLKO). Lentiviral particles were either prepared as previously described (37) or obtained from the RNAi Platform (Broad Institute). Transduction and subsequent selection on 2 μg/ml puromycin dihydrochloride (Sigma-Aldrich) were performed as described previously (69). From day 12 or 13 to day 18, cells were cocultured on a murine MS-5 stromal cell layer at a cell density of 3 × 10^5^ to 6 × 10^5^ cells/ml as described previously (70, 71). On day 18, cells were replated on a fresh MS-5 stromal cell layer. Cells were harvested on either day 17 or 18 or day 20 and passed through a 5-μm Supor filter (Pall) to remove residual nucleated cells. Filtered, enucleated cells were stored at 4°C in incomplete RPMI (RPMI 1640 [Sigma-Aldrich] supplemented with 25 mM HEPES and 50 mg liter^−1^ hypoxanthine) until use in subsequent experiments.

### Flow cytometry-based measurement of erythrocyte receptor expression.

Erythrocytes were washed three times in phosphate-buffered saline (PBS)–3% bovine serum albumin (BSA) blocking buffer, pelleted in a 96-well plate at 5 × 10^5^ erythrocytes per well (cultured erythrocytes) or 1 × 10^6^ erythrocytes per well (peripheral erythrocytes), and finally resuspended in 50 μl (cultured erythrocytes) or 100 μl (peripheral erythrocytes) of PBS–3% BSA or the appropriate antibody solution. The following antibodies were used at the indicated dilutions: phycoerythrin (PE)-conjugated anti-DARC (1:10) (Miltenyi Biotec), anti-CD71-PE (1:10) (Miltenyi Biotec), fluorescein isothiocyanate (FITC)-conjugated anti-GPA (1:50) (clone 2B7; Stem Cell Technologies), anti-GPC-FITC (1:500) (BRIC 10; Santa Cruz), anti-band 3-FITC (1:100) (BRIC 6-FITC; International Blood Group Reference Laboratory [IBGRL]), anti-BSG (1:1,000) (clone MEM-M6/6; Axxora [Exbio]), anti-CR1 (1:200) (Santa Cruz), anti-DAF (1:3,000) (BRIC 216; IBGRL), anti-RhD (1:20) (BRAD3-FITC; IBGRL), and anti-GPA/B (1:8,000) (clone E3; Sigma-Aldrich). To measure GPB expression, erythrocytes were treated with 1 mg/ml trypsin (Sigma-Aldrich) to remove GPA before incubation in the GPA/B antibody. For measurement of GPA and GPC expression in Senegal, the following probes were used in addition to the aforementioned antibodies: for anti-GPA, BRIC 256 IgG (IBGRL; 1:100,000) and BRIC 256-FITC Fab fragment (1:20); for anti-GPC, BRIC 10 IgG (IBGRL; 1:500,000), BRIC 10-FITC Fab fragment (1:100), and BRIC 4 IgG (IBGRL; 1:8,000). Fab fragments were produced from whole IgG by papain digestion (Pierce Fab preparation kit; Thermo Scientific) and FITC labeled (ProtOn fluorescein labeling kit; Vector Laboratories), followed by extensive dialysis in PBS.

Cells were incubated for 1 h at room temperature and washed three times in blocking buffer. Unstained cells and cells stained with directly conjugated antibodies were resuspended in 100 μl PBS for analysis on a MACSQuant flow cytometer (in Boston [Miltenyi Biotec]) or a BD FACSCalibur (in Senegal [BD Biosciences]). Erythrocytes incubated with all other antibodies were then incubated in anti-mouse IgG-Alexa Fluor 488 (1:1,000; Life Technologies) for 30 min at room temperature. Control samples with no prior antibody incubation were incubated in either anti-mouse IgG2a-PE (1:10; Miltenyi Biotec) or anti-mouse IgG-Alexa Fluor 488. Cells were washed twice and subjected to flow cytometric analyses. The data were analyzed using FlowJo 4 v. 10.0.7 for flow cytometry done in Boston or FlowJo v. 8.8.6 for flow cytometry done in Senegal.

### Quantitative cell surface proteomics.

pLKO control, GPA KD, GPB KD, and GPC KD cultured erythrocytes (cRBCs) were prepared as described above. The following sets of cells were labeled by aminooxy-biotin as described previously (72, 73): pLKO cRBCs, 10.2 × 10^6^ cells (in duplicate); GPA KD cells, 16.2 × 10^6^ cells; GPB KD cells, 3.5 × 10^6^ cells; GPC KD cells, 11.2 × 10^6^ cells; and peripheral RBCs, 10.0 × 10^6^ cells (in duplicate).

Briefly, surface sialic acid residues were oxidized with sodium meta-periodate (Thermo Scientific) and then biotinylated with aminooxy-biotin (Biotium). The reaction was quenched, and the biotinylated cells were incubated in a 1% Triton X-100 lysis buffer. Biotinylated glycoproteins were enriched with high-affinity streptavidin-agarose beads (Pierce) and washed extensively. Captured protein was denatured with dithiothreitol (Sigma-Aldrich), alkylated with iodoacetamide (IAA) (Sigma-Aldrich), and digested on-bead with trypsin (Promega) in 200 mM HEPES (pH 8.5) for 3 h. Tryptic peptides were collected and labeled using tandem mass tag (TMT) reagents (Thermo Scientific). The reaction was quenched with hydroxylamine and TMT-labeled samples were combined in a 1:1:1:1:1:1:1 ratio. Labeled peptides were enriched and desalted, and then 6% of the total sample was subjected to mass spectrometry.

Mass spectrometry data were acquired using an Orbitrap Fusion coupled with an UltiMate 3000 Nano LC (Thermo Scientific). Peptides were separated on a 75-cm PepMap C_18_ column (Thermo Scientific). Peptides were separated using a 180-min gradient of 3 to 33% acetonitrile in 0.1% formic acid at a flow rate of 200 nl/min. Each analysis used a MultiNotch MS3-based TMT method (72, 73). The scan sequence began with an MS1 spectrum (Orbitrap analysis; resolution of 120,000, 400 to 1,400 Th, automatic gain control [AGC] target of 2 × 10^5^, and maximum injection time of 50 ms). MS2 analysis consisted of collision-induced dissociation (CID) (quadrupole ion trap analysis; AGC of 15,000, normalized collision energy [NCE] of 35, and maximum injection time of 120 ms). The top 10 precursors were selected for MS3 analysis, in which precursors were fragmented by higher-energy collisional dissociation (HCD) prior to Orbitrap analysis (NCE of 55, maximum AGC of 2 × 10^5^, maximum injection time of 150 ms, isolation specificity of 0.5 Th, and resolution of 60,000).

### Sample collection for *ex vivo* invasion assays and erythrocyte receptor expression.

Collection of clinical isolates and their experimental use were approved by the Ethics Committee of the Ministry of Health in Senegal and by the Institutional Review Board of the Harvard T.H. Chan School of Public Health. Sample collection was done in November 2013, toward the end of the transmission season in Senegal. After informed consent from patients presenting with uncomplicated malaria in Thies, Senegal, ∼4 ml of whole blood was collected from each patient in sodium citrate Vacutainers and transported to Dakar, Senegal. Samples arrived in the laboratory within 6 h of draw, and after washes in incomplete RPMI and removal of the buffy coat, a fraction of parasitized cells was treated with Vibrio cholerae neuraminidase (Sigma-Aldrich), chymotrypsin (Worthington), and trypsin and placed in culture conditions until invasion assay setup.

For measurement of erythrocyte receptor expression in healthy Senegalese, 2 to 3 ml of whole blood was collected from healthy donors from the Senegalese National Blood Transfusion Center in Dakar, Senegal. Flow cytometry was performed on the day of blood collection.

### Parasite cultures.

P. falciparum cultures were maintained in human O^+^ erythrocytes. Parasites were grown at 2% hematocrit (HCT) in complete RPMI medium (incomplete RPMI supplemented with 2.57 mM sodium bicarbonate [Sigma-Aldrich], 0.25% AlbuMAX II [Life Technologies], plus 0.25% AB^+^ serum [for *ex vivo* cultures] or 0.5% AlbuMAX II [for laboratory strains and the short-term culture-adapted strain]). Cultures were kept at 37°C in a modulator incubator chamber gassed with 1% O_2_, 5% CO_2_, and 94% N_2_.

### Enzyme treatments.

Ring-stage parasite cultures were washed three times in incomplete RPMI and treated with 1 mg/ml trypsin, 1 mg/ml chymotrypsin, and 66.7 mU/ml of Vibrio cholerae neuraminidase for 1 h at 37°C with gentle mixing. Enzyme-treated cells were then washed twice in incomplete RPMI and once in complete RPMI and then resuspended at 2% HCT in complete RPMI and placed in culture.

### Invasion assays.

Invasion assays were performed in half-area 96-well plates in duplicate or triplicate. At the late trophozoite or schizont stage, the parasite culture was resuspended and adjusted to 0.5% HCT based on hemocytometer cell counts (for *ex vivo* invasion assays in Senegal) or based on volume (for invasion assays conducted in Boston). GPA, GPB, and GPC KD cultured erythrocytes (cRBCs) and pLKO cRBCs were adjusted to 0.5% HCT based on hemocytometer cell counts. Parasitized cells were added to cRBCs at an 80:20 ratio (Fig. 4B). For *ex vivo* invasion assays in Senegal, 10 μl of the total sample volume of 40 μl was used for smears to determine the starting parasitemia by light microscopy. Boston-based invasion assays were performed with a total sample volume of 30 μl. Assays were harvested at 30 to 40 h after setup by making smears or cytospin preparations, which were stained with May-Grünwald and Giemsa stains to determine the final parasitemia by light microscopy. Five hundred to 2,000 erythrocytes were counted, depending on the experiment. The invasion efficiency based on final parasitemia was determined for each strain after normalization to pLKO control cells.

### Statistical analyses.

Univariate analyses were performed using GraphPad Prism v. 5.0 for Mac OS X. Significant differences between each knockdown group and control group were determined using a one-way analysis of variance (ANOVA) and Dunnett's multiple-comparison test.

### Data analysis for quantitative surface proteomics.

Mass spectra were processed using a Sequest-based in-house software pipeline as previously described (74). Data were searched using the human UniProt database (April 2014) concatenated with common contaminants and filtered to a final protein-level false-discovery rate of 1%. Proteins were quantified by summing TMT reporter ion counts across all peptide-spectral matches using in-house software as previously described (74), excluding peptide-spectral matches with poor-quality MS3 spectra (a combined signal/noise ratio of less than 250 across all TMT reporter ions). For protein quantitation, reverse and contaminant proteins were removed.

A subset of 78 membrane proteins were identified based on the presence of criteria extracted from the UniProt (75) database (type I/II/III/IV transmembrane domain, multipass transmembrane domain, glycosylphosphatidylinositol [GPI] anchored, or lipid anchored) or on predictions of transmembrane helices based on the TMHMM 2.0 program (76, 77). Each reporter ion channel was summed across all 78 proteins and normalized assuming equal protein loading across all samples. To compare the relative abundances of the membrane proteins between the pLKO cRBCs and KD cRBCs, proteins with a coefficient of variation of >0.25 in the pLKO technical replicates were excluded from further analysis.

The fold change for each protein was calculated as the ratio of the signal/noise ratio for GPA/B/C KD to the average signal/noise ratio for pLKO controls. *P* values (significance A) were calculated using Perseus v. 1.5.1.6 and adjusted with the Benjamini-Hochberg method (78). To compare the abundance of plasma membrane proteins between pLKO cRBCs and peripheral RBCs, for each protein the normalized signal for each reporter channel was renormalized to a total of 1, and the data were clustered using the k-means algorithm in Cluster v. 3.0 with a Euclidean distance metric (79) and subsequently displayed using TreeView v. 1.1.6r4 (80).

## Supplementary Material

Supplemental material
