# Supplementary material for: Genetic Evidence for Erythrocyte Receptor Glycophorin B Expression Levels Defining a Dominant Plasmodium falciparum Invasion Pathway into Human Erythrocytes
Source: Infect Immun. 2017 Sep 20;85(10):e00074-17. doi: 10.1128/IAI.00074-17 (PMC5607420; doi:10.1128/IAI.00074-17)
Supplement: Supplemental material [file supp_85_10_e00074-17__index.html]

Supplemental material 

# Genetic Evidence for Erythrocyte Receptor Glycophorin B Expression Levels Defining a Dominant Plasmodium falciparum Invasion Pathway into Human Erythrocytes

## Supplemental material

- Supplemental file 1 -

  Fig. S1. Characterization of glycophorin A-, B-, and C-depleted cultured erythrocytes. Table S2. Description of proteins with significant fold change in abundance. Table S3. Table of neuraminidase sensitivity of *P. falciparum* strains.

  PDF, 476K
- Supplemental file 2 -

  Table S1. Description and analysis of all proteins identified on peripheral erythrocytes and cultured erythrocytes by quantitative surface proteomics.

  XLSX, 113K
