# Supplementary material for: Genetic Evidence for Erythrocyte Receptor Glycophorin B Expression Levels Defining a Dominant Plasmodium falciparum Invasion Pathway into Human Erythrocytes
Source: Infect Immun. 2017 Sep 20;85(10):e00074-17. doi: 10.1128/IAI.00074-17 (PMC5607420; doi:10.1128/IAI.00074-17)
Supplement: Supplemental material [file IAI.00074-17_zii999092172s1.pdf]

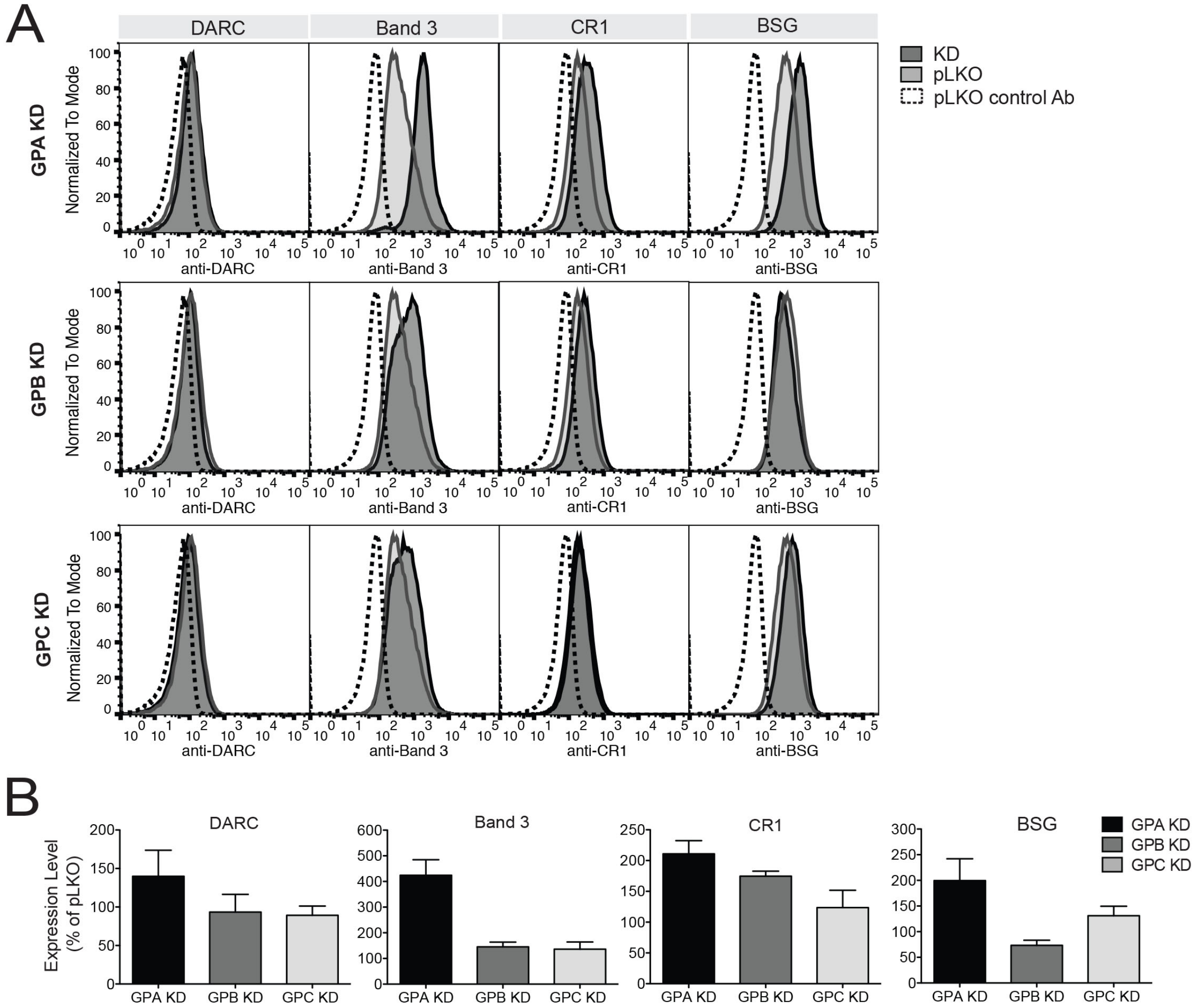

FIGURE S1. Characterization of glycophorin A-, B- and C-depleted cultured erythrocytes. A. Expression of *P. falciparum* invasion receptors (CR1 and BSG), band 3 and *P. vivax* receptor DARC on the surface of GPA KD, GPB KD and GPC KD cRBCs as determined by flow cytometry. Representative plots are shown. **B.** Mean expression ± standard deviation of *P. falciparum* invasion receptors (CR1 and BSG), DARC and band 3 receptors on the surface of GPA KD, GPB KD and GPC KD cRBCs as determined by flow cytometry from two to four experiments. cRBCs were passed through a 5 μm filter to remove nucleated cells prior to flow cytometry.

TABLE S2. Description of proteins with significant fold change in abundance.

cRBCs	Protein	Change	*P*-value	Description	Function/ Role
GPA KD	PARK7	Decreased	0.0141	Protein DJ-1	Positive regulator of androgen receptor-dependent transcription.
GPB KD	BCAM	Decreased	0.0312	Basal Cell Adhesion Molecule, Lutheran Blood Group	Member of the immunoglobulin superfamily. Receptor for the laminin extracellular matrix protein.
GPB KD	RHAG	Decreased	0.0418	Ammonium transporter Rh type A	Transport of ammonium and carbon dioxide across the erythrocyte membrane.
GPB KD	BSG	Decreased	0.0474	Basigin, Ok Blood Group	Essential role in *P. falciparum* invasion of erythrocytes.
GPB KD	SLC7A5	Increased	0.0381	Large neutral amino acids transporter small subunit 1	Transport of large neutral amino acids.
GPB KD	ATP12A	Increased	0.0336	Isoform 2 of Potassium-transporting ATPase alpha chain 2	ATPase, H+/K+ transporter.
GPC KD	SLC30A1	Increased	0.0464	Zinc transporter 1	Transport of sugars, bile salts and organic acids, metal ions and amine compounds.
GPC KD	CD46	Increased	1.8441x 10^{7}	Complement Membrane Cofactor Protein	Complement regulatory protein.

TABLE S3. Table of neuraminidase sensitivity of *P. falciparum* strains.

Strain	IE into Nm pRBCs[a]	Resistant/Sensitive[b]	Refs
Dd2	N/A	S	(1)
3D7	N/A	R	(2)
3D7ΔEBA175	N/A	R	(2)
3D7ΔRh2b	N/A	R	(3)
7G8	N/A	R	(4)
HB3	N/A	R	(1)
Sen51	50.8 ± 8.8	S	This Study
Th266	17.3 ± 7.5	S	This Study
Th268	51.1 ± 6.3	S	This Study
Th303	45.3 ± 11.4	S	This Study
Th304	105.6 ± 4.6	R	This Study
Th305	48.9 ± 7.6	S	This Study
Th306	14.1 ± 3.1	S	This Study
Th312	61.4 ± 22	R	This Study

[a]Invasion efficiency into neuraminidase-treated (Nm) pRBCs relative to untreated pRBCs; based on final parasitemia. Parasitemia was determined by SYBR green flow cytometry. Mean ± standard deviation (Sen51, Th266, Th268) or range (Th303, Th304, Th305, Th306, Th312) for one biological replicate.

[b]Sensitivity to neuraminidase treatment for field isolates in this study was based on a cutoff of 60%.

References

1. Dolan SA, Proctor JL, Alling DW, Okubo Y, Wellems TE, Miller LH. 1994. Glycophorin B as an EBA-175 independent Plasmodium falciparum receptor of human erythrocytes. Mol Biochem Parasitol 64:55-63.
2. Duraisingh MT, Maier AG, Triglia T, Cowman AF. 2003. Erythrocyte-binding antigen 175 mediates invasion in Plasmodium falciparum utilizing sialic acid-dependent and -independent pathways. Proc Natl Acad Sci U S A 100:4796-801.
3. Duraisingh MT, Triglia T, Ralph SA, Rayner JC, Barnwell JW, McFadden GI, Cowman AF. 2003. Phenotypic variation of Plasmodium falciparum merozoite proteins directs receptor targeting for invasion of human erythrocytes. Embo J 22:1047-57.
4. Hadley TJ, Klotz FW, Pasvol G, Haynes JD, McGinniss MH, Okubo Y, Miller LH. 1987. Falciparum malaria parasites invade erythrocytes that lack glycophorin A and B (MkMk). Strain differences indicate receptor heterogeneity and two pathways for invasion. J Clin Invest 80:1190-3.
